# Using Passive Sensing to Isolate a Biosignature for Craving Among Individuals in Early Alcohol Use Disorder Recovery

**DOI:** 10.1111/adb.70182

**Published:** 2026-08-10

**Authors:** Sara Mei, Noah N. Emery, David Eddie

**Affiliations:** ^1^ Department of Psychology Colorado State University Fort Collins Colorado USA; ^2^ Center for Addiction Medicine, Massachusetts General Hospital Recovery Research Institute Boston Massachusetts USA; ^3^ Department of Psychiatry Harvard Medical School Boston Massachusetts USA

## Abstract

Craving is one of the most robust proximal predictors of both treatment dropout and relapse during early recovery for alcohol use disorder (AUD). Craving can onset rapidly, and the ability to accurately detect or modulate cravings varies significantly within‐ and between‐persons. Because of this, craving management is a central feature of most current AUD treatment models. However, impaired interoceptive awareness, which is common in early AUD recovery, can stymie individuals' capacity to implement craving management strategies. Measurement and detection of craving through passive biosensor monitoring could support novel just‐in‐time adaptive interventions that can mitigate this vulnerability. This study sought to characterize the biosignatures of craving through the measurement of heart rate variability (HRV) using a combination of ambulatory electrocardiogram (ECG) monitoring and ecological momentary assessment (EMA) in individuals in the first year of a current AUD recovery attempt (*N =* 40, observations = 400). A multilevel negative binomial regression analysis was conducted to estimate correlations between craving and HRV. Results demonstrated that, at the within‐person level, high‐frequency HRV (HF‐HRV; *IRR =* 1.01, *p* = 0.013) and SD1 (*IRR =* 0.97, *p* = 0.020) were associated with craving at the same moment. Findings highlight HRV as a viable target for passive monitoring in interventions aimed at supporting craving mitigation in early AUD recovery, particularly at the momentary level.

## Graphical abstract

This study sought to characterize the biosignatures of craving through the measurement of heart rate variability (HRV) using a combination of ambulatory electrocardiogram (ECG) monitoring and ecological momentary assessment (EMA) in individuals in the first year of a current AUD recovery attempt (*N* = 40, observations = 400). A multilevel negative binomial regression analysis demonstrated that, at the within‐person level, high‐frequency HRV (HF‐HRV; *IRR* = 1.01, *p* = 0.013) and SD1 (*IRR* = 0.97, *p* = 0.020) were associated with craving at the same moment.

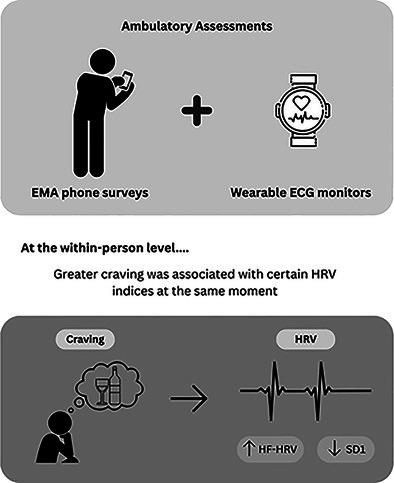

## 1 Introduction

Alcohol use disorder (AUD) ranks among the most detrimental mental disorders in the United States, contributing to approximately 178 000 deaths annually [1] and exacting an economic toll of $249 billion each year [2]. This burden is attributable to critical treatment gaps as well as the relapsing nature of addiction [3, 4]. Empirically supported behavioural treatments for AUD are initially efficacious in helping people to stop or reduce alcohol use. However, the risk of relapse remains, particularly within the first year of recovery [5]. This vulnerability during the initial recovery stage underscores the need for novel interventions that can mitigate this relapse risk.

Craving is a cardinal feature of substance use disorder (SUD), including AUD, and linked to the initiation, maintenance, recurrence of substance use and treatment disengagement [6, 7]. Among individuals admitted to SUD treatment, craving emerges as a particularly salient predictor of relapse risk, demonstrating an inverse relationship with time to posttreatment relapse [8], independent of pretreatment substance use patterns [9]. This risk is further compounded by the fact that individuals in early recovery typically exhibit poor interoceptive awareness of affective states and impaired affect regulation [10]; these deficits compromise capacity to implement craving management strategies, particularly given that craving onsets rapidly [11] and craving intensity fluctuates considerably both within individuals and across the population [12]. Taken together, these findings underscore a critical need for interventions that target craving management, especially those that simultaneously address the limited emotional awareness that characterizes early AUD recovery.

Just‐in‐time adaptive interventions (JITAIs) leveraging real‐time data from mobile devices and sensors have emerged as a promising strategy to support recovery from AUD. In theory, a JITAI could detect high‐risk moments and deliver timely, tailored support when it is most needed. This approach holds particular promise in addressing the dynamic and fluctuating nature of craving in daily life and may have utility as a complement to existing first‐line addiction treatments. And yet, to date, there has been limited research exploring the biomarkers of craving that could be leveraged to drive such a JITAI. Ideal physiological targets would be those that can be easily measured using existing wearable technology so that any developed JITAI could be implemented on devices already in use, thereby maximizing accessibility and scalability. Heart rate (HR) and heart rate variability (HRV) are ideal targets for passive monitoring because they can be reliably measured using photoplethysmograph sensors ubiquitously embedded in smartwatches and fitness trackers.

HRV, defined as the variation in time between consecutive heartbeats, is a well‐established indicator of autonomic nervous system activity [13] and is a known indicant of affective states like craving [14, 15]. In individuals with SUD, alternations in trait‐level HRV have been observed compared to those without SUD, suggesting that consistent disruptions in autonomic regulation may be linked to addictive behaviours and attendant states [16]. In laboratory cue‐reactivity paradigms, HRV is a robust predictor of craving [17] and changes in affect [18] in response to substance‐related cues. Studies have also shown that craving is associated with changes in HR and HRV during withdrawal phases, potentially reflecting the stress and autonomic dysregulation experienced by those in early recovery [19, 20].

Target HRV indices should focus on robust indicators of parasympathetic activity, given the established role of the parasympathetic nervous system (PNS) in emotion regulation and putative link to craving [19]. Accordingly, the present study focused on three widely used indices of PNS functioning: high‐frequency heart rate variability (HF‐HRV), a frequency domain index reflecting respiratory‐linked vagal modulation of heart rate [21]; the percentage of adjacent normal‐to‐normal intervals differing by more than 50 ms (pNN50), a time‐domain measure of vagal activity [22]; and SD1, the standard deviation of points perpendicular to the line of identity on a Poincaré plot, which indexes short‐term parasympathetic regulation [23]. With the current constellation of indices, we explored a parasympathetic measure from each HRV analytical class—frequency‐domain (HF‐HRV), time‐domain (pNN50) and non‐linear (SD1)—to provide convergent yet nonredundant measures of parasympathetic function and capture the multifaceted role of the PNS in emotion and craving regulation.

This measurement strategy was further complemented by pairing wearable biosensor technologies with ecological momentary assessments (EMA; [24]), which afford real‐time data collection in naturalistic environments and enable us to capture the dynamic temporal processes underlying craving as they unfold. EMA circumvents many methodological constraints of laboratory‐based research while illuminating moment‐to‐moment associations between psychophysiological states and alcohol use. Despite the potential of combining EMA and biosensor data to elucidate affective and psychophysiological mechanisms of substance use, few studies have integrated these methodologies to date.

The current study sought to examine if moments of high craving are characterized by acute changes in target HRV indices for individuals in their first year of AUD recovery. Based on previous models that demonstrate the multidimensional and multifaceted nature of craving, we hypothesized that moments of high craving would be associated with changes in HRV concurrently (i.e., at the same moment). Specifically, in line with previous research [19], we hypothesized that at the within‐person level, self‐reported craving would be positively associated with HF‐HRV, such that moments of increased craving would be associated with higher levels of HF‐HRV. Additionally, based on previous work [20], we hypothesized that at the between‐person level, HF‐HRV will be inversely associated with craving, such that people with lower overall levels of HF‐HRV will exhibit higher overall craving. Though pNN50 and SD1 tend to be highly correlated with HF‐HRV, because these measures have been less well explored in the context of craving identification, we treated these measures as exploratory.

## 2 Methods

### 2.1 Participants

The current study analysed secondary EMA and electrocardiogram (ECG) data (*N =* 40; EMA observations = 400) collected as part of a parent study examining cognitive and physiological aspects of affect in individuals in early recovery from AUD (AA025251; PI: Eddie; see [25] for primary outcomes). Inclusion criteria were (1) meeting past‐year AUD criteria (based on the DSM‐5 criteria; [6]), (2) endorsing a current goal of alcohol abstinence, (3) being in the first year of a current AUD recovery attempt and (4) participating in outpatient AUD treatment (e.g., outpatient, mutual‐help programmes and individual therapy) or a mutual‐help programme (e.g., Alcoholics Anonymous). Given acute alcohol withdrawal could impact HRV measurements, participants were required to have ≥ 2 weeks of alcohol and other drug abstinence, assessed using the timeline follow‐back, before enrollment to minimize the influence of physiological withdrawal symptoms. Exclusion criteria were (1) having cardiac arrhythmias or serious medical conditions that may affect HRV (e.g., cardiovascular disease), (2) taking medications that directly influence HRV (e.g., beta blockers) and (3) having an active SUD related to a drug other than alcohol in the past year (also based on DSM‐5 criteria). The analytic sample (*N* = 40) was 60.0% male, aged 22 to 65 (*M* = 42.2, *SD* = 13.0). Participants were 75.0% White/European American, 17.5% Black/African American, 5.0% Asian and 2.5% other race/mixed race.

### 2.2 Procedure

Procedures were approved by the institutional review board (Mass General Brigham IRB# 2016P001178). Participants completed a phone screening to determine eligibility. Eligible participants completed an intake appointment with baseline measures and were trained to use the EMA application on their personal smartphone. Participants then completed 4 days of EMA monitoring using the MetricWire smartphone application [26]. The programme generated three prompts for participants to complete brief ~2‐min assessments about participants' experience at random times within three, 3‐h blocks (e.g., 10 AM–1 PM, 2–5 PM, and 7–10 PM). In addition to random surveys, participants were instructed to self‐initiate a survey in moments when they felt high levels of stress, alcohol craving, or felt at risk for alcohol use. To encourage random EMA survey completion, participants were given a $30 bonus for completing ≥ 90% of the surveys.

### 2.3 Measures

#### 2.3.1 Baseline Measures

**Alcohol use.** At baseline, alcohol use was assessed with Alcohol Dependence Scale (ADS; [27]) and timeline follow‐back (TLFB; [28]). The ADS is a 29‐item measure that evaluates the severity of alcohol dependence based on symptoms and behaviours associated with AUD. The measure has demonstrated high internal consistency (*α* = 0.92; [27]). The TLFB is considered the gold standard for assessing alcohol use by asking individuals to provide an estimate of their daily drinking patterns over a specified time period. In the current study, participants were asked to report their alcohol consumption for the 30 days prior to baseline.

#### 2.3.2 EMA Measures

**Craving.** During random and self‐initiated begin use reports, craving was assessed with a single item of urge to use alcohol on an 11‐point scale from 0 (*no craving*) to 10 (*extreme craving*). This measure is widely used in laboratory and EMA research [29, 30, 31] and previous EMA work supports the criterion validity [32]. EMA survey timestamps were used to align craving reports with ECG data.

Substance use related outcomes are relatively low‐base rate at the moment level [33, 34]. This produces datasets that are often highly skewed with many zeroes. This issue is even further exacerbated by intensive repeated assessments inherent to event‐level studies [33]. Thus, given the single item for craving consisted of nonnegative integer responses and exhibited substantial positive skew (skewness = 1.09) as well as a large proportion of zero values (39.5%), craving was treated as a count with a negative binomial reference distribution to increase interpretability.

**HRV.** Ambulatory measures of HRV were measured using an eMotion Faros 180 ambulatory ECG monitor and accelerometer, an FDA 510(k) cleared, ce marked Class IIa medical device (see [25] for review of full detail of physiological recording and control measure check). Participants wore the device around their chest and affixed using an elastic belt embedded with two ECG contact points under the pectoral muscles. Participants were then instructed to wear the device throughout the day while engaging in their normal daily activities, allowing for continuous ECG recording. Sequences of heart beat‐to‐beat intervals (RRI) were recorded and exported to Kubios HRV software [35], and 5‐min HRV segments corresponding to EMA assessments were extracted for analysis and calculation of HRV indices and mean heart rate. HRV indices were derived from 5‐min ECG windows aligning with the EMA surveys (i.e., the 5‐min window immediately preceding the survey timestamp to avoid capturing any effects of participants physically completing the surveys). For self‐initiated surveys, the same procedure was applied. After cubic interpolation of the nonequidistant waveform, the RRI sequence was manually checked for artefacts and irregular beats in Kubios (i.e., missed, misplaced or ectopic beats) and edited where necessary. Minimal filtration was applied only when indicated.

Heart rate, expressed as beats per minute, was derived by calculating the average number of R‐spikes in the ECG signal occurring each minute during the 5‐min recording period. HRV indices were calculated from edited sequential R‐R intervals derived from the ECG signal. HF‐HRV was calculated using Fourier analysis [36, 37] and defined as spectral power in the 0.15–0.40‐Hz band. pNN50 is the percent of adjacent normal‐to‐normal intervals that differ from each other by more than 50 ms. SD1 represents the standard deviation of beat‐to‐beat variability perpendicular to the line of identity in a Poincaré plot [23]. To calculate individual participants' mean HRV across the ECG monitoring period, an average score for each HRV index was calculated for each participant.

### 2.4 Planned Analysis

After checking for skew and kurtosis, outlying values, determined by *SD* > 3.29, *p* < 0.001 as per standard convention (see [38] for review), were changed to one unit greater than the nearest nonoutlying value. Two observations were changed to one unit greater than the nearest nonoutlying value (i.e., winsorized). We centered indices of HRV by subtracting person averages from momentary values at the within‐level. At the between‐level, variables were centered by subtracting the overall sample averages from person‐level averages (grand‐mean centered). Person‐centered variables reflect moment‐to‐moment deviations from one's average level, and grand‐mean centered variables reflect person deviations from the overall average for the sample. Centering also partially normalizes the distributions, which simulation studies suggests are optimal for within‐person effects and superior to global transformations (i.e., logarithmic transformations; [39]).

To test the hypothesized concurrent associations between momentary HRV indices and craving in real‐time using EMA, we estimated a multilevel negative binomial regression model with random intercepts and an unstructured variance–covariance matrix using Stata 18. Craving was specified as the outcome variable with a negative binomial reference distribution. Importantly, all variables were assessed contemporaneously. Therefore, analyses estimated associations among variables at the same moment and does not support conclusions regarding causality or temporal ordering. Craving was modelled as the outcome variable to facilitate a consistent examination of its associations with the range of HRV indices included in the analyses. This approach additionally permitted estimation of each HRV indicator's unique association with craving while controlling for the other HRV indices and covariates included in the model (i.e., semipartial estimates). The negative binomial reference distribution was selected because it accommodates positively skewed count outcomes and permits meaningful zeros responses while providing flexibility, given alcohol‐related outcomes such as craving often have relatively low‐base rates [33]. The data had a two‐level structure in which moments (Level 1 [L1]; within‐person) were nested within‐persons (Level 2 [L2]; between‐persons). Multilevel models account for the nonindependence of observations that results from the nesting of time‐varying observations via momentary assessments within persons (L1). Multilevel models are also robust to unequal numbers of observation per participant. Slopes were fixed rather than random because the primary aim lies in estimating the overall effect rather than exploring between‐person variability. Moreover, incorporating random slopes would introduce additional model complexity not central to the research question. The models contained within‐person momentary indices of HRV (i.e., HF‐HRV, pNN50 and SD1) associated with craving at the same moment (L1) and person‐level averages of HRV indices associated with average craving over the sampling period (L2).

L1 covariates included day‐of‐the‐week and day‐in‐the‐study to control for daily variation in substance use and mood as well as time‐dependent change in variable frequency [40]. L2 covariates included sex and age to account for potential sex and age differences in patterns of substance use [41, 42] and HRV [43]. The inclusion of momentary and average indicators allowed for isolation of the within‐person associations of state fluctuations in the association between craving and HRV from between‐person associations. Distinctions between person‐average craving and HRV from momentary states (e.g., a moment of high craving or HRV) capitalized on the full complement of the EMA data by distinguishing dynamic, within‐person changes in momentary craving or HRV from between‐person, individual differences in typical (i.e., dispositional) craving or HRV level. In short, craving and HRV were expected to vary not only within each person over time (L1; within‐person) but also on average from person to person (L2; between‐persons). An intercept‐only model (without any predictors) estimated intraclass correlations (ICCs) (i.e., variability in predictors attributed to between‐person effects relative to within‐person influences).

### 2.5 Statistical Power and Minimum Detectable Effect Sizes

To determine the minimum detectable effect sizes in our data, we conducted a computer simulation using the Monte Carlo feature of Mplus 8.5 [44]. Consistent with previous research on affect and substance use (e.g., [45, 46]), 50% of the variance in craving and HRV was specified at the within‐person level. The focal effects of interest in this study are the effects of momentary HRV indicators on momentary craving. Given previous research has not examined these associations using EMA methods, we conducted multiple Monte Carlo simulations with small effect sizes ranging from *β* = 0.05 to *β* = 0.35 for both within‐ and between‐level associations. Results using 10 000 replications indicated a sample of 40 individuals with 10 observations each would be ≥ 0.80 powered to detect within‐person effects of *β* = 0.15 or higher and between‐person effects of *β* = 0.33 or higher.

## 3 Results

### 3.1 Descriptive Statistics

Over the 4‐day EMA + HRV monitoring period, participants completed 313 random surveys (*M* = 7.8, *SD* = 2.8) and 87 self‐initiated surveys (*M* = 5.4, *SD* = 6.2) over the sampling period, totaling to 400 completed surveys. Table 1 includes descriptive statistics for L1 and L2 variables of interest. Given that participants completed 313 random surveys out of 480 total random surveys, the compliance was 65.0%. This level of compliance is consistent with rates reported in previous EMA studies involving individuals who meet criteria for AUD and other SUD diagnoses [47]. Out of 40 participants, 25 completed self‐initiated surveys (*M* = 11.4, *SD* = 4.1). Across the 400 observations included in the analyses, participants contributed an average of 10.0 observations (range = 2–21) over the 4‐day period. We note that the large range in number of surveys completed across participants are expected in EMA studies and are accommodated by multilevel models, which do not require an equal response distribution per participant and use all available data while accounting for the nesting of observations within individuals. Participants reported an average of 12% drinking days (*SD* = 18.9) over the 30 days preceding study enrollment. Participants' mean ADS was 23.8 (*SD* = 8.5) indicating a ‘substantial level’ of AUD severity [27]. Approximately 61.5% of observations of craving were 1 or greater (*M* = 2.2, *SD* = 2.60).

**TABLE 1:** Descriptive statistics for Level 1 and Level 2 variables.

	*M*	*SD*
Craving	2.22	2.60
HF‐HRV	275.13	469.33
pNN50	7.24	11.71
SD1	17.39	12.99

*Note: N =* 40 individuals, observations = 400. *M =* mean, *SD* = standard deviation. HF‐HRV = high‐frequency heart rate variability; pNN50 = percent of adjacent normal‐to‐normal cardiac interbeat intervals greater than 50 ms; SD1 = the standard deviation of points perpendicular to the line of identity on the Poincaré plot.

ICCs for focal variables estimate the proportion of variance attributable to within‐person versus between‐person differences. The ICCs were craving = 0.38, HF‐HRV = 0.35, pNN50 = 0.51 and SD1 = 0.52. This suggests that 38% of the total variance explained by craving was due to stable between‐person differences, while 62% was attributable to within‐person fluctuations over time. HF‐HRV demonstrated a moderate ICC, indicating notable within‐person variability, consistent with its role as a physiological marker that responds dynamically to momentary changes in autonomic regulation. In contrast, HRV‐related indices such as pNN50 and SD1 exhibited higher ICCs, suggesting a relatively greater proportion of variance attributable to stable individual differences.

### 3.2 Multilevel Modelling

A multilevel negative binomial regression model was estimated to test the hypothesized momentary associations of HRV with craving.^1^ At the within‐person level (L1), consistent with our hypothesis, higher‐than‐average levels of HF‐HRV were associated with more instances of craving (*IRR =* 1.01, *p* = 0.013, 95% CI [1.001, 1.002]). Additionally, SD1 was inversely associated with craving, such that moments of higher‐than‐average levels of SD1 were associated with lower incidents of craving (*IRR =* 0.970, *p* = 0.020, 95% CI [0.945, 0.995]). Momentary pNN50 was not significantly associated with craving (*p* = 0.264).

At the between‐person level (L2), no significant effects for person‐level indicators of HRV were found (*p*s ≥ 0.05; see Table 2). The model included a random intercept for participants (variance = 1.32, SE *=* 0.41), indicating meaningful between‐person variability in craving after accounting for covariates.

**TABLE 2:** Multilevel model analysis for HRV indicators on craving.

	IRR	SE	*z*	*p*
Within‐person (Level 1; L1)				
**HF‐HRV**	**1.01**	**< 0.01**	**2.50**	**0.** **013**
pNN50	1.02	0.01	1.12	0.264
**SD1**	**0.97**	**0.01**	**−2.32**	**0.** **020**
Monday	1.22	0.37	0.66	0.510
Tuesday	1.74	0.56	1.73	0.084
Wednesday	1.45	0.44	1.23	0.221
Thursday	1.29	0.32	1.03	0.303
**Friday**	**1.74**	**0.38**	**2.54**	**0.** **011**
**Saturday**	**1.81**	**0.36**	**2.96**	**0.** **003**
Day‐in‐the‐study	1.12	0.07	1.79	0.073
Between‐persons (Level 2; L2)				
HF‐HRV	1.00	< 0.01	−0.73	0.467
pNN50	0.92	0.09	−0.86	0.392
SD1	1.13	0.10	1.38	0.167
Sex	1.79	0.74	1.41	0.157

*Note: N =* 40 individuals, observations = 400. IRR = incident rate ratio, SE = standard errors. HF‐HRV = high‐frequency heart rate variability; pNN50 = percent of adjacent normal‐to‐normal cardiac interbeat intervals greater than 50 ms; SD1 = the standard deviation of points perpendicular to the line of identity on the Poincaré plot. Orthogonal day‐of‐the‐week indicators represent that day's effect compared with the reference day, Sunday. Sex = sex assigned at birth (0 = *male*, 1 = *female*).

## 4 Discussion

Craving is a complex, multifaceted phenomenon central to AUD and AUD treatment [6], but accurately detecting it in real time remains a challenge due to its individualized and dynamic nature [12]. Although passive HRV biosensors have shown potential in lab settings, their real‐world applicability has been underexplored. The current study explored real‐time, ambulatory associations between craving and HRV. We found that momentary craving was associated with acute changes in HF‐HRV and SD1, providing preliminary evidence that physiological markers of cravings can be detected in naturalistic settings. Although the study limitations constrain some generalizability regarding real‐world implementation, these findings serve as a proof of concept to support the potential utility of biosensor‐based approaches for ambulatory assessments of craving.

Consistent with hypotheses, higher‐than‐average levels of HF‐HRV were positively associated with greater craving in the same moment. This aligns with previous laboratory research, where craving was found to be positively associated with HF‐HRV for both individuals with AUD in short‐term and long‐term abstinence from alcohol [19]. Craving and emotion regulation have been examined using emotionally evocative stimuli within emotion induction procedures—however, most have been limited to stress‐ and/or craving‐related cues [19]. The pattern of effects found in the present study extends this previous work with ecologically valid, ambulatory data. HF‐HRV is considered a marker of parasympathetic activity [48], higher levels of which are linked to greater attention, cognitive performance, and better self‐regulation [49]. This aligns with contemporary models of craving that suggest that craving is not merely a conditioned response but involves higher order cognitive and emotional processes, consistent with the operationalization of craving as a multidimensional construct that engages both automatic and controlled processes [50]. The increase in HF‐HRV observed during moments of craving may reflect individuals' conscious or unconscious attempts to regulate affective and physiological responses to urges to use alcohol and other drugs. This aligns with cue‐reactivity research, which has demonstrated that HRV increases when individuals exert effort to suppress or manage craving‐related thoughts and emotions [17].

Interestingly, although HF‐HRV was positively associated with craving at the momentary level, SD1 showed an inverse association, with lower‐than‐average SD1 observed during elevated craving. This divergence likely reflects distinct temporal dynamics of parasympathetic modulation rather than conflicting physiological signals. Although both HF‐HRV and SD1 are influenced predominantly by parasympathetic activity, they capture distinct aspects of heart rate dynamics. HF‐HRV reflects respiratory‐linked vagal modulation of heart rate (i.e., respiratory sinus arrhythmia), whereas SD1 indexes short‐term, beat‐to‐beat variability in cardiac intervals. The divergent associations of HF‐HRV and SD1 with craving suggest that craving may not be characterized by a simple increase or decrease in parasympathetic activity. Instead, craving may involve distinct changes across different features of cardiac autonomic regulation. One possibility is that, as craving intensifies, individuals become increasingly focused on craving‐related thoughts, sensations and urges, whereas their cardiovascular system exhibits reduced beat‐to‐beat variability. In this framework, heightened craving may be associated with greater engagement with the craving experience alongside a reduced flexibility in short‐term autonomic regulation. Future research is needed to clarify the mechanisms underlying this pattern and its relevance to substance use behaviour.

At the between‐person level, person‐average HF‐HRV was not significantly associated with person‐average craving levels. This divergence between within‐person and between‐person effects is noteworthy. Although momentary fluctuations in HF‐HRV tracked concurrent craving, stable individual differences in average HRV were not predictive of overall craving vulnerability. This pattern suggests that HF‐HRV's relationship to craving is primarily dynamic and state‐dependent rather than reflecting trait‐level parasympathetic capacity. The lack of a between‐person association contrasts with laboratory research linking higher trait HF‐HRV to better emotion regulation and reduced reactivity to substance cues [19], highlighting a key distinction between controlled experimental settings and the heterogeneous demands of naturalistic environments. In real‐world contexts, craving vulnerability may be less determined by overall parasympathetic capacity and more dependent on moment‐to‐moment psychophysiological responding to contextual triggers and situational demands. Future research should examine whether the between‐person HF‐HRV‐craving relationship emerges only under specific contextual conditions or whether additional person‐level factors (e.g., coping strategies, environmental stressors, and substance use patterns) moderate this association.

This study had numerous strengths, including the use of ambulatory assessment, enhancing ecological validity by capturing craving experiences in naturalistic settings. Additionally, the use of multilevel modelling allowed for a more nuanced understanding of HRV/craving associations by accounting for within‐ and between‐person variability, providing deeper insights into the complex relationship between these constructs. Despite these strengths, several limitations must be noted. First, as this study focused specifically on individuals with AUD, there is limited generalizability to others in different recovery contexts. Findings should be replicated in more diverse SUD samples to determine if the HRV/craving relationship holds across different populations and contexts. Second, some researchers have suggested that using absolute (e.g., ‘extremely’) versus relative (e.g., ‘the strongest urge I have ever experienced’) anchor‐point labels for craving items may lead to differences in self‐reports of craving depending on individual interpretation [51]. Relatedly, despite previous research showing a single item with absolute anchors exhibits strong criterion validity [32], utilizing a single‐item craving measure may have inadequately captured the various semantic dimensions of craving that individuals may use to describe their anecdotal experiences [52]. Third, ambulatory HRV is sensitive to many physiological and behavioural factors, such as physical activity, sleep–wake processes, medication use, and other daily activities. At the same time, the primary aim of the current study was to explore HRV/craving associations in a naturalistic setting rather than the traditional laboratory setting so that HRV measurements might reflect physiological functioning across the diverse contexts encountered in everyday life. Although these factors may contribute additional variability to HRV measurements, the significant associations observed between HRV indices and craving suggest that these relationships may be robust across naturally occurring situations. In other words, the present findings should be interpreted as reflecting potential HRV and craving associations in real‐world environments rather than craving‐specific physiological effects. Future research should incorporate these variables as time‐varying covariates to better isolate the contribution of craving in autonomic processes. Lastly, the analyses were cross‐sectional and not causal. Thus, the observed same‐moment associations do not support conclusions regarding causality or temporal ordering.

The present findings establish the feasibility of passive HRV monitoring via wearable biosensors for real‐time craving detection in naturalistic settings. Importantly, establishing concurrent links between passive HRV indices and craving is a critical first step. Future work should pursue two complementary directions. First, prospective analyses examining lagged HRV‐craving associations could clarify the temporal dynamics and predictive utility of these relationships; specifically, whether HRV changes precede craving, follow craving onset, or if HRV and craving changes happen in tandem. If HRV changes precede craving, an early warning system could be possible. The EMA survey approach used here was structured such that there were several hours apart precluding these types of analyses. Future works would benefit from higher density EMA sampling to test these models. Second, integrating HRV biomarkers with other physiological and behavioural signals (e.g., movement patterns, sleep, and social context) could enhance detection specificity and move toward clinically actionable algorithms. Once validated, such multimodal approaches could be embedded in mobile health applications or JITAI to enable real‐time detection and intervention on craving states. This trajectory from real‐world monitoring to personalized, precision‐guided treatment represents a significant opportunity to enhance addiction recovery by shifting from reactive to proactive intervention strategies.

## 5 Conclusions

This study examined whether moments of elevated craving are characterized by distinct parasympathetic HRV signatures among individuals in early AUD recovery using concurrent biosensor and ecological momentary assessment data. Momentary HF‐HRV showed a positive association with craving, whereas SD1 showed an inverse association, suggesting that parasympathetic dynamics at different timescales reflect distinct physiological processes during craving. These findings provide preliminary evidence that HRV biomarkers, particularly those capturing parasympathetic function, can be detected in real‐world contexts and may serve as indices of acute craving states.

The absence of a significant between‐person association between baseline HF‐HRV and overall craving levels highlights an important distinction: parasympathetic capacity appears to be most relevant as a dynamic, momentary process rather than as a stable trait predictor of vulnerability. If replicated, these findings suggest that HRV‐based algorithms could enable real‐time craving detection and inform JITAI tailored to individual physiological signatures. Such an approach would represent a meaningful advance toward precision addiction treatment, allowing clinicians to deliver timely, personalized support at moments of highest vulnerability and potentially reducing relapse risk and its associated harms.

At the same time, the present findings should be viewed as an early step toward this goal rather than evidence that HRV‐informed JITAIs are ready for clinical deployment. The current study extends prior laboratory‐based work by demonstrating associations between HRV and craving in individuals in recovery and provides preliminary evidence that these relationships may also be detectable in everyday settings. Further research is needed to characterize these associations with greater precision using longer monitoring periods, more frequent ecological assessments, and larger samples. Future studies should also incorporate lagged analyses to clarify the temporal ordering of changes in HRV and craving, address potential confounding influences, and examine more homogeneous recovery cohorts to reduce between‐person heterogeneity. Replication in larger and more controlled studies will be essential for determining the robustness, generalizability and clinical utility of HRV‐based approaches for monitoring and intervening on craving in real‐world recovery contexts.

## Author Contributions

**Sara Mei:** conceptualization, data curation, formal analysis, writing – original draft, writing – review and editing. **Noah N. Emery:** conceptualization, data curation, formal analysis, supervision, writing – original draft, writing – review and editing. **David Eddie:** funding acquisition, methodology, investigation, project administration, resources, conceptualization, supervision, writing – review and editing.

## Funding

This research was supported by the National Institute on Alcohol Abuse and Alcoholism award F32AA025251.

## Conflicts of Interest

Senior Author D.E. is on the scientific advisory boards of mental‐healthcare companies ViviHealth and Innerworld and is a partner in Peer Recovery Consultants. The remaining authors declare that they have no known potential competing financial or personal interests.

## Data Availability

The data that support the findings of this study are available from the corresponding author upon reasonable request.

## Endnotes

^1^
This model was also estimated as a linear multilevel model, which yielded the same pattern and magnitude (i.e., *z‐*scores) of significant and nonsignificant effects.
